# Association between arterial hypertension and nutritional status in adolescents from Goiânia, Goiás, Brazil

**DOI:** 10.1371/journal.pone.0188782

**Published:** 2017-12-18

**Authors:** Mayara Maria Souza de Almeida, Rafael Alves Guimarães, Paulo César Brandão Veiga Jardim, Ana Luiza Lima Sousa, Márcia Maria de Souza

**Affiliations:** 1 Faculty of Nursing, Federal University of Goiás, Goiânia, Goiás, Brazil; 2 Institute of Tropical Pathology and Public Health, Federal University of Goiás, Goiânia, Goiás, Brazil; 3 Faculty of Medicine, Federal University of Goiás, Goiânia, Goiás, Brazil; Escola Paulista de Medicina, BRAZIL

## Abstract

**Introduction:**

Adolescents are a population with unique lifestyle challenges, including physical inactivity, inadequate nutrition, and obesity, all of which increase the risk of developing hypertension (HTN). The objective of this study has been to estimate the prevalence of factors associated with hypertension in adolescents in the city of Goiânia City, Central Brazil.

**Methods:**

Between 2013and2014, a cross-sectional population study on cardiovascular risk in adolescents, was conducted with the participation of 1,586 adolescents in 108 classes at 36 schools (public and private) in Goiânia city. All of the adolescents were interviewed to establish their sociodemographic and behavioral characteristics related to hypertension and nutritional status. Anthropometric and blood pressure data were collected following a protocol. A Poisson regression, stratified by gender, was used to verify the factors associated with HTN.

**Results:**

In this mixed-gender group of 1,586 students, the prevalence of HTN was 6.2% (95% CI: 4.6–8.2%) in girls and 14.0% (95% CI: 10.2–18.8%) in boys—about twice as high in boys as in girls (p <0.001). Obesity was independently associated with HTN in both genders. Being overweight was a risk factor for HTN. In addition, there was a positive correlation between the SBP/SBP percentile and the BMI Z-score/Nutritional status (NS)in both genders. A high prevalence of physical inactivity was also observed in the adolescents investigated, especially in the girls. On the other hand, more boys than girls were found to be obese.

**Conclusion:**

The results of this investigation revealed the need for strategies to prevent and control HTN and its risk factors, especially in Brazil's schools. In addition to the constant surveillance of HTN prevalence and risk factors (in particular, being overweight or obese), information should be distributed to promote beneficial health behaviors among adolescents.

## Introduction

In 2012, cardiovascular diseases accounted for about 17.5 million deaths each year worldwide, with arterial hypertension (HTN) causing more than half of the deaths (9.4 million)[1]. HTN also accounted for 45% of deaths from heart disease and 51% of deaths from stroke at a global level [2]. In 2008, approximately 40.0% of adults aged 25 years or over had a diagnosis of HTN. In fact, the number of people with HTN has increased from 600 million in 1980 to 1 billion in 2008 [2]. In general, the prevalence of HTN is higher in high-income countries than in those with lower levels of economic development (35.0% versus 40.0%) [2].

In the Americas, 35.0% of adults are estimated to have HTN [2]. In Brazil, a national multicenter study entitled the “National Health Survey (*Pesquisa Nacional de Saúde* [PNS]),”conducted in 2013, estimated a self-reported prevalence of 21.4% (95.0% confidence interval [95.0% CI]: 20.8–22.0%) in the adult population of the country [3]. In 2016, the study, “Surveillance of Risk and Protection Factors for Chronic Diseases via a Telephone Survey (*Vigilância de Fatores de Risco e Proteção para Doenças Crônicas por Inquérito Telefônico* [VIGITEL])”found a 25.7% prevalence of HTN (95.0% CI: 24.9–26.5%) in Brazilian adults [4].

An increasing number of Brazilian adolescents has HTN [5]. In general, HTN occurs asymptomatically in adolescence, which makes early diagnosis difficult [6]. In adolescents, HTN is measured using blood pressure (BP) and weight, height, and age percentiles, as established by the international guidelines of “The Fourth Report on the Diagnosis, Evaluation, and Treatment of High Blood Pressure in Children and Adolescents” (2004) [7]. A systematic review of studies published up to 2008 involving adolescents aged between10–20 from all regions of Brazil (without representation of the North region), estimated that the prevalence of HTN was 8.1% (95.0% CI: 6.2–10.5%) and higher in boys (8.7%; 95.0% CI: 5.8–13.0%) than in girls (6.3%, 95.0% CI: 4.4–9.0%)[8]. Another review of studies published up to 2012 involving young people aged 10–19 found a similar prevalence: (8.0%; 95.0% CI: 5.0–11.0%), 9.3% (95.0% CI: 5.6–13.6%) in boys and 6.5% 20 (95.0% CI: 4.2–9.1%) in girls [5]. However, in both reviews, only one study covered the Central-West region of Brazil, which includes the States of Goiás, Mato Grosso, and Mato Grosso do Sul, as well as the Federal District/Brasilia) [9]. That study, conducted using 2,118 adolescents from Goiânia city (Goiás State), found that2.9% of the adolescents had HTN(95.0% CI: 2.4–3.7%) [9].

HTN is a complex and multifaceted disease. Many factors contribute to the high prevalence rates of HTN among adolescents[10].The non-modifiable risk factors associated with HTN in this population are representative, mainly involving a family history of HTN or cardiovascular disease [7], male gender[11–13],socioeconomic status, and race/skin color[10]. The modifiable risk factors include the use of legal drugs (e.g. alcohol and tobacco), physical inactivity [14, 15], unhealthy eating habits [10, 16], the presence of comorbidities (e.g. diabetes and hypercholesterolemia), and poor sleep quality and/or short sleep duration [10, 17].

The modifiable risk factors of inadequate diet and physical inactivity lead to central adiposity, overweight, and obesity [10]. Overweight and obesity correspond substantially to cases of HTN and have increased significantly worldwide, especially in adolescents[18]. It is estimated that thousands of deaths occur due to the consequences of being overweight or obese, through diabetes, musculoskeletal disorders, cancer and HTN [19]. Worldwide, by 2014, more than 1.9 billion adolescents aged 13–18 or over were overweight, and 600 million were obese [19]. A systematic review has shown that the prevalence of excessive weight in children and adolescents from developing countries is 12.9% in males and 13.4% in females. In developed countries, this frequency increases to 23.8% in boys and 22.6% in girls[20]. In Brazil, the prevalence of excessive weight in adolescents is estimated to be21.7% in boys and 19.4% in girls [21].

Chronic diseases such as HTN have caused great concern, both to the scientific community and to society as a whole, since they lead to higher and higher mortality rates worldwide, among adolescents as well as adults. Adolescents present a unique lifestyle, often involving multiple risk behaviors for HTN, including physical inactivity and inadequate nutrition. In addition, many adolescents are overweight or obese. In Brazil, studies of the prevalence and determinants of HTN are scarce, especially in the Center-West region of the country. Most of the existing studies were carried out in the Southeast region, the most developed area of the country. The identification of potential risk factors in this population is an important tool in the development of measures to prevent and control HTN and its consequences. The object of this study, therefore, is to estimate the prevalence and factors associated with HTN in adolescents in Goiânia City, Central Brazil.

## Materials and methods

This study is part of a larger project entitled “Study of Cardiovascular Risks in Adolescents (*Estudo de Riscos Cardiovasculares em Adolescentes”*[ERICA]), a cross-sectional multicenter study funded by the Brazilian Ministry of Health to estimate the prevalence of risk factors for cardiovascular diseases in adolescent students aged 12–17 in Brazil. The study was conducted among schools in Brazilian cities with more than 100,000 inhabitants between 2013 and 2014. The methods used in this national study have been described in more detail in previous publications [22, 23]. The data analyzed for the present study came from Goiânia City, the capital of Goiás State (in the Central-West Region of Brazil), which has approximately1.296 million inhabitants.

Included in the study were: (i) Adolescents between 12 and 17 years of age, within the age range defined for adolescence (12–18 years) adopted by the Statute of the Child and Adolescent of Brazil (*Estatuto da Criança e do Adolescente do Brasil*) [24]; (ii)Students enrolled in morning or afternoon classes (known as “terms”); (iii) Students in the last three grades of elementary school or the three grades of high school, attending public or private schools in Goiânia City [25].The following groups were excluded: (i) pregnant students, and (ii) those with physical or mental disabilities, whether temporary or permanent[22, 26].

During the first stage of the study, schools were selected using probability proportional to size sampling (PPS). The size of each school was equal to the ratio between the number of students in its eligible classes and its distance from Goiânia City (the state capital).The PPS selection was conducted after sorting school records by census location (urban or rural) and school governance (private or public) [22, 23]. This study selected 36 schools in Goiânia.

During the second stage, three classes with equal probabilities were selected from each school, using the class year as an age proxy. A worksheet was used to select the participating classes. It was specific to each school sampled, and used for class and student selection [22, 23]. In Goiânia, 108 classes were selected.

During the third stage, all of the students in the selected classes were invited to participate in an examination composed of interviews, anthropometric analyses, and BP measurements [22, 23]. In Goiânia, 3,217 potentially eligible adolescents were selected.

Three questionnaires were used (one for the adolescents, the second or their parents, and the third for the school). Anthropometric and blood pressure data were also collected. The adolescent questionnaire was self-administered, using a personal digital assistant (PDA) [22, 23]. This study used only the students’ self-completed questionnaires.

Systolic blood pressure (SBP) and Diastolic blood pressure (DBP) were measured using a digital monitor (Omron 705-IT, Omron Healthcare Inc., Lake Forest, Illinois, USA), validated for use on adolescents [27]. BP was measured using a cuff appropriately sized for a teenager’s right arm, while each student was seated, with his or her feet on the floor. Three consecutive measurements were made for each student, with a three-minute interval between them. The first measure was discarded and the mean of the last two measurements was used. The anthropometric measures used in the present study were weight and height. Weight was verified using an electronic scale (Lider®, Rosele Araçatuba, São Paulo,Brazil) with a capacity of 200 kg and a variation of 50g. Height was measured using a portable stadiometer (Alturexata®, Belo Horizonte, Minas Gerais, Brazil), with a resolution of 1 mm and a range of up to 213 cm [22, 25].

All measurements were made by a single healthcare professional (a nurse or nutritionist) [22, 25].The field team was trained prior to the start of the study and supervised at all stages of the study. During data collection, all measurements were subject to quality control, in which the data were analyzed to check for any problems in the procedures carried out by interviewers, technicians, or information processors. Procedural manuals and videos were prepared to train the team to administer the anthropometric evaluation and BP measurements, so as to guarantee the standardization of the procedures and minimize measurement errors [22, 23, 25].

### Variables

#### Dependent variable

HTN is measured using weight, height and age percentiles, as established by “The Fourth Report on the Diagnosis, Evaluation, and Treatment of High Blood Pressure in Children and Adolescents” (2004)[7].Adolescents were classified as normotensive (normal BP) when their BBP and DBP were below the 90th percentile. They were prehypertensive when their SBP or DBP were between the 90th and 95th percentiles, or when they had an SBP greater than or equal to 120 mmHg or a DBP greater than or equal to 80, with a percentile lower than 95. They were hypertensive when either their SBP or their DBP presented a value equal to or greater than that found in the 95th percentile [7, 22]. In this study, the dependent variable was HTN (no or yes).

#### Independent variables

The following independent variables were analyzed:

1. Sociodemographics: age (years). The students were divided into two age groups: 12–14 and 15–17[26], and categorized by their race/color skin, as self-reported by participants. The ethnic group classifications were as follows:white, black, mixed race, or other (including native Brazilian and Asian). These reflected the typical racial classifications used for the Brazilian population[14];type of school (public or private); and term (morning or afternoon)[25].
2. Tobacco and alcohol consumption: lifetime tobacco use (no or yes), defined as experimentation or any cigarettes smoked ever in their lives-even one or two puffs; current tobacco use (no or yes), defined as the use of tobacco at least once during the previous 30 days [26]; lifetime alcohol use (no or yes); current alcohol use (no or yes), defined as the consumption of at least one glass of any alcoholic beverage during the previous 30 days[28]; binge drinking in the previous 30 days (no or yes), defined as the consumption of five or more doses of alcohol on a single occasion for men, and four or more doses on a single occasion for girls[29].
3. Practice of physical activity: adolescents were classified as active or inactive. A Self-Administered Physical Activity Checklist was used to measure theirlevels of physical activity[30]. This instrument consisted of a list of 24 physical activities; it allowed the adolescent to report the frequency (days) and the time (hours and minutes) spent practicing physical activities during the previous week [30, 31]. The time and frequency of all of the activities listed were added together to calculate the total number of minutes spent. Adolescents who did not accumulate at least 300 minutes/week of physical activity were considered inactive [31].
4. Medical health history: diabetes mellitus (no or yes), defined as a medical diagnosis and/or the use of diabetes medications; hypercholesterolemia (no or yes), defined as a medical diagnosis and/or the use of medications for hypercholesterolemia.
5. Nutritional status (NS): Body mass index (BMI), defined as weight (Kg) divided by the square of height (cm). NS was calculated using World Health Organization reference curves and the BMI index for age, grouped by gender. The cut-off points adopted were: Z-score <-3 (very low weight); Z score ≥ -3 and <-2 (low weight); Z-score ≥ -2 and ≤ 1 (normal); Z-score> 1 (overweight) and ≤ 2; Z-score> 2 (obesity)[22, 32].To analyze the factors associated with HTN, we excluded adolescents with very low weight and low weight.

### Statistical analysis

The data were analyzed using the STATA program, version 14.0. In order to estimate the associations and their 95.0% CI, complex sample routines were used in the STATA, using “survey” package [33].Categorical variables were presented in relative frequencies and continuous variables on average in the descriptive analysis, accompanied by 95.0% CI. Student's t-test t or Pearson’s chi-square test was used to compare the variables between the genders.

To verify the relationship between NS and BP, we used the Pearson’s correlation coefficient (r) [34, 35]. Correlations were made between the following variables: NS and SBP percentiles, NS and DBP percentiles, the BMI Z-score and SBP percentile, and the BMI Z-score and DBP percentile. Analyses were carried out on each sample, the total sample, and by gender. To verify the magnitude of the correlations by gender, correlations found between the genders were also compared, using the “Cocor” software R package [36].

To verify the factors associated with HTN, bivariate and multivariable analyses were performed. Only variables with a value of p < 0.20 and those with potential confounding and adjustment (age, race/skin color, school type, and term) were included in the Poisson regression models [37, 38]. Analyses were stratified by gender. Values of p < 0.05 were considered statistically significant.

### Ethical considerations

This study was approved by the Ethics Committee of the Federal University of Rio de Janeiro (protocol n° 007/2009) and the Ethics Committee of the Clinics Hospital of the Federal University of Goiás (protocol n° 007/2010). The parents and/or guardians of the adolescents consented to their participation by signing a free and informed consent form. In addition, the adolescents provided their own written consent by signing the study consent form.

## Results

**Fig 1:**
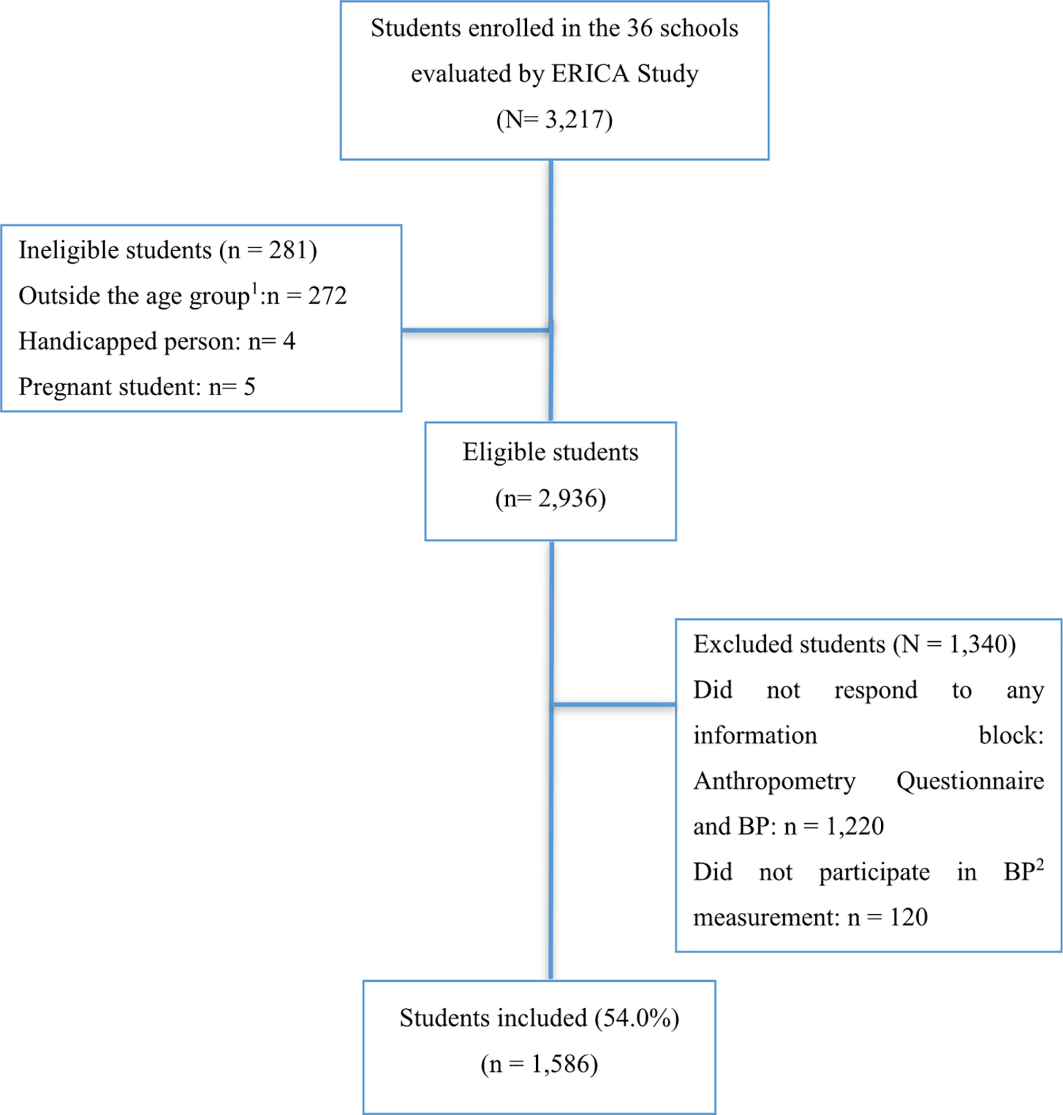
Flow chart for selection of study participants, ERICA Study, Goiânia, Goiás, Central-West region of Brazil, 2013–2014 Legend: ^1^Children under 12 and over 17 years of age; ^2^blood pressure.

Fig 1 shows the flowchart for selecting study participants. Of the total number of students enrolled in the 36 Goiânia schools evaluated during the ERICA Study (n = 3,217), 281 (8.7%) were considered ineligible (272 were outside the age group [younger than 12 or older than 17], four were disabled, and five were pregnant). Of the eligible students (n = 2,936), 1.340 (45.6%) failed to respond to any information block or to participate in the BP measurement. Thus, 1,586 adolescents aged between 12 and 17 (973 females and 613 males) ultimately participated in the study.

Table 1 provides a descriptive analysis of the variables (other than nutritional status, BP, and HTN) categorized by gender. Of the total number of participants, approximately half were between 12 and 14 years old (50.7%) and self-declared as mixed race (51.3%). The adolescents interviewed were predominantly from public schools (72.9%) and the morning term (81.2%). There was no difference between the genders regarding sociodemographic characteristics (p > 0.05).

**Table 1:** Descriptive analysis of variables (except NS, BP, and HTN prevalence), according to gender. ERICA study, Goiânia, Goiás, Central-West region of Brazil, 2013–2014.

Variables	Total (N = 1,586)		Male (N = 613)		Female (N = 973)		p^1^
	%	95.0% CI^2^	%	95.0% CI^2^	%	95.0% CI^2^	
**Age (years)**							
12–14	50.7	(50.7;50.7)	50.1	(50.1;50.1)	51.3	(51.3;51.3)	1.000
15–17	49.3	(49.3;49.3)	49.8	(48.8;48.8)	48.7	(48.7;48.7)	
**Race/skin color (self-reported)**							
White	38.7	(33.0; 44.7)	44.5	(34.3; 55.3)	32.6	(28.1; 37.5)	0.051
Black	6.9	(5.1; 9.3)	8.0	(5.1; 12.3)	5.9	(3.4; 10.1)	
Mixed	51.3	(45.3; 57.2)	44.9	(35.9; 54.2)	57.8	(53.5; 62.0)	
Other^3^	3.1	(2.4; 4.0)	2.6	(1.6; 3.2)	3.7	(2.8; 4.8)	
**Type of school attended**							
Public	72.9	(54.6; 85.8)	73.6	(54.4; 86.7)	72.3	(54.3; 85.1)	0.593
Public	27.1	(14.1; 45.4)	26.4	(13.3; 45.6)	27.7	(14.9; 45.7)	
**Term**							
Morning	81.2	(64.2; 91.3)	82.5	(65.2; 92.2)	80.0	(62.6; 90.5)	0.260
Afternoon	18.8	(8.7; 35.8)	17.5	(7.8; 34.8)	20.0	(9.5; 37.3)	
**Tobacco use (lifetime)**							
No	80.4	(77.7; 83.7)	80.7	(75.5; 85.1)	80.9	(77.1; 84.2)	0.951
Yes	19.2	(16.3; 22;3)	19.3	(14.9; 24.5)	19.1	(15.8; 22.8)	
**Tobacco use (previous 30 days)**							
No	95.5	(93.6; 96.9)	94.6	(91.6; 96.6)	96.4	(94.3; 97.7)	0.151
Yes	4.5	(3.1; 6.4)	5.4	(3.4; 8.4)	3.6	(2.2; 5.7)	
**Alcohol use (lifetime)**							
No	43.0	(39.8; 46.2)	45.2	(40.7; 49.8)	40.7	(37.4; 44.1)	0.062
Yes	57.0	(53.8; 60.2)	54.8	(50.0; 59.3)	59.3	(55.9; 62.6)	
**Alcohol use (previous 30 days)**							
No	76.5	(73.1; 79.6)	75.8	(72.0; 79.2)	77.2	(72.3; 81.4)	0.583
Yes	23.5	(20.4; 26.9)	24.2	(20.8; 28.0)	22.8	(18.6; 27.7)	
**Binge drinking(previous 30 days)**							
No	94.1	(92.6; 95.3)	95.0	(92.5; 96.6)	93.2	(90.7; 95.1)	0.277
Yes	5.9	(4.7; 7.4)	5.0	(3.3; 7.5)	6.8	(4.9; 9.3)	
**Physical activity**							
Active	52.7	(47.6; 57.7)	64.7	(57.5; 71.4)	40.3	(36.0; 44.7)	**< 0.001**
Inactive	47.3	(42.3; 52.4)	35.3	(28.6; 42.5)	59.7	(55.3; 64.0)	
**Diabetes mellitus (self-reported)**							
No	96.5	(95.2; 97.4)	97.4	(95.4; 98.6)	95.5	(93.7; 96.8)	0.085
Yes	3.5	(2.6; 4.8)	2.6	(1.4; 4.6)	4.5	(3.2; 6.3)	
**Hypercholesterolemia (self-reported)**							
No	90.1	(88.6; 92.9)	91.2	(87.4; 92.9)	90.7	(88.4; 92.6)	0.770
Yes	9.0	(7.1; 11.4)	8.8	(6.1; 12.6)	9.3	(7.4; 11.6)	

^1^Pearson’s chi-square test

^2^95.0% Confidence Interval

^3^Includes Native Brazilian and Asian ethnic groups.

Regular consumption of tobacco and alcohol, as well as binge drinking during the previous 30 days, were reported by 4.5%, 23.5% and 5.9% of participants, respectively. Approximately half (47.3%) of the participants were considered insufficiently active. A higher proportion of girls than boys reported insufficient physical activity (59.7% versus 35.3%; p < 0.001). The self-reported prevalence of diabetes mellitus and hypercholesterolemia came to 33.5% and 9.0%, respectively (Table 1).

Table 2 synthesizes the anthropometric measures, BP (SBP and DBP),the BP percentile distribution, and HTN prevalence by gender. The means of weight and height were 57.1 kg and 164.0 cm, respectively. Weight and height were statistically higher in boys than in girls (p <0.001). In addition, the BMI Z-score global mean was 0.20, and this mean was statistically higher in boys than in girls (0.20 versus 0.12; p = 0.037). The prevalence of overweight and obesity came to 17.1% and 8.9%, respectively. Boys were more likely to be obese than girls (12.6% versus 5.1%, p <0.001).

**Table 2:** Descriptive analysis of the variables NS, BP, BP percentile, and HTN prevalence according to gender. ERICA Study, Goiânia, Goiás, Central-West region of Brazil, 2013–2014.

Variables	Total (N = 1,586)	Male (N = 613)	Female (N = 973)	p
Weight (kg)	57.2 (56.0–58.3)	60.2 (58.3; 62.1)	54.1 (53.2–53.9)	**< 0.001**^1^
Height (cm)	164.0 (163.2–164.7)	167.4 (166.1; 168.8)	160.4 (160.0–160.7)	**< 0.008**^1^
BMI (kg/m^2^)	21.0 (20.8–21.4)	21.2 (20.8; 21.7)	21.0 (20.7–21.2)	0.592^1^
BMI z-score	0.20 (0.12; 0.28)	0.28 (0.16; 0.40)	0.12 (0.04; 0.20)	**0.037**^1^
Nutritional status				
Very underweight/underweight	2.5 (1.6; 4.0)	2.7 (1.5; 4.7)	2.4 (1.3; 4.3)	**< 0.001**^2^
Normal	71.4 (68.9; 73.9)	66.4 (62.7; 69.9)	76.6 (73.2; 79.8)	
Overweight	17.1 (14.7; 19.8)	18.3 (14.7; 22.5)	15.9 (13.3; 18.9)	
Obese	8.9 (7.1; 11.1)	12.6 (9.4; 16.6)	5.1 (3.7; 7.0)	
SBP (mmHg)	110.1 (109.1–112.0)	114.4 (112.8; 115.9)	107.5 (106.6–108.5)	**< 0.001**^1^
SBP percentile				
< 50th	47.1 (42.5; 51.8)	40.0 (33.6; 46.8)	54.4 (49.8; 59.1)	**< 0.001**
50th	40.2 (36.5; 43.9)	43.0 (28.0; 48.1)	37.2 (33.1; 41.5)	
90th	4.5 (3.3; 6.1)	3.6 (2.0; 6.5)	4.5 (3.2; 6.1)	
95th	5.6 (3.7; 8.5)	8.8 (5.2; 14.8)	2.3 (1.5; 3.5)	
99th	2.6 (1.7; 4.0)	4.5 (2.8; 7.1)	1.5 (0.7; 3.1)	
DBP (mmHg)	66.1 (65.2–67.0)	66.3 (65.2–67.4)	66.0 (65.0–66.9)	0.417^1^
DBP percentile				
< 50th	37.8 (32.8; 42.9)	35.5 (29.3; 42.4)	40.0 (34.6; 45.6)	0.602^2^
50th	54.9 (49.6; 60.1)	57.3 (50.0; 64.3)	52.3 (47.0; 57.6)	
90th	3.9 (2.8; 5.4)	3.8 (2.1; 6.8)	4.0 (2.8; 5.7)	
95th	2.6 (1.9; 3.6)	2.6 (1.5; 4.4)	2.7 (1.7; 4.4)	
99th	0.8 (0.4; 1.7)	0.9 (0.4; 2.0)	0.7 (0.3; 2.0)	
BP Classification				
Normal	77.6 (75.7–79.8)	68.5 (64.4–72.3)	86.4 (83.5–88.9)	**< 0.001**^2^
Prehypertension	12.5 (10.1–15.5)	17.5 (23.8–22.0)	7.4 (5.6–9.7)	
HTN	10.1 (8.2–12.4)	14.0 (10.2–18.8)	6.2 (4.6–8.2)	

^1^Student's t-test

^2^Pearson’s chi-square test.

The SBP mean was 110.1 mmHg and the DBP mean was 66.1 mmHg. Boys had a higher SBP than girls (114.4 mmHg versus 107.5 mmHg, p < 0.001). There was also a difference between the genders in the distribution of SBP percentiles (p < 0,001). However, there was no difference between boys and girls in relation to the means of DBP and the DBP percentile. Of the total number of adolescents, 77.3% (95.0% CI: 75.0–79.8%) and 12.6% (95.0% CI: 10.1–15.5%) presented with normotension and prehypertension, respectively. The prevalence of HTN was 10.1% (95% CI: 8.2–12.4%). HTN prevalence in girls and boys was 6.2% (95.0% CI: 4.6–3 8.2%) and 14.0% (95.0% CI: 10.2–18.8%), respectively (Table 2). Boys had a prevalence 2.26 times higher than girls (Crude PR: 2.26, 95.0% CI: 1.39–3.66, p = 0.002).

In the correlation analysis, we observed a very weak positive correlation between the BMI Z-score and the SBP percentile (*r* = 0.325; p < 0.001) and between the BMI Z-score and the DBP percentile (*r* = 0.176; p < 0.001) in the total sample. Considering the correlation analysis between the NS and BP percentiles, the results were similar (*r* = 0.313; p < 0.001 between the NS and SBP percentile and *r* = 0.182; p < 0.001 between the NS and SBP) (Table 3).

**Table 3:** Correlation between the SBP/DBP percentile,the BMI Z-score, and NS, according to gender. ERICA study, Goiânia, Goiás, Central-West region of Brazil, 2013–2014.

Variables	SBP percentile		DBP percentile	
	r^1^	p	r^1^	p
All				
BMI Z-score	0.325	**< 0.001**	0.176	**< 0.001**
NS	0.313	**< 0.001**	0.182	**< 0.001**
Female				
BMI Z-score	0.273	**< 0.001**	0.148	**< 0.001**
NS	0.266	**< 0.001**	0.185	**< 0.001**
Male				
BMI Z-score	0.373	**< 0.001**	0.216	**< 0.001**
NS	0.346	**< 0.001**	0.212	**< 0.001**

^1^Pearson coefficient correlation.

An analysis of the stratified correlation for females showed a very weak positive correlation between the BMI Z-score and the DBP percentile (*r* = 0.148; p < 0.001) and a weak positive correlation between the BMI Z-score and the SBP percentile (r = 0.273; p < 0.001). NS values for girls were similar (*r* = 0.185; p = 0.001 between the NS and DPB percentiles and *r* = 0.266; p < 0.001 between the NS and SBP percentiles). For boys, a weak positive correlation was observed between the BMI Z-score and the DBP percentile (*r* = 0.216; p < 0.001), and between the BMI Z-score and the SBP percentile (*r* = 0.373; p < 0.001). NS values for boys were similar (*r* = 0.212; p < 0.001 between the NS and DBP percentiles and *r* = 0.346; p < 0.001 between the NS and SBP percentiles) (Table 3). The correlation between the SBP percentile and the BMI Z-score in boys was statistically stronger than the correlation for girls (p = 0.03), which did occur in the case of the BMI Z-score and the DBP (p = 0.187). The correlation between the DBP percentile and NS, and between the SBP percentile and NS were similar between the genders (p > 0.05).

Tables 4 and 5 show the prevalence and potential factors associated with HTN in female and male adolescents, respectively. In this analysis, NS was the only risk factor for HTN in both genders. Table 6 shows the Poisson regression model of factors associated with HTN by gender. It was found that, after adjustment, being overweight (APR: 2.28, 95.0% CI: 1.26–4.09) was independently associated with HTN in female adolescents. Obesity was a common risk factor for boys (APR: 2.30, 95.0% CI: 1.09–4.84) and girls (APR: 3.48; 95.0% CI: 1.42–8.54).

**Table 4:** Prevalence and potential risk factors associated with HTN in adolescent females. ERICA Study, Goiânia, Goiás, Central-West region of Brazil, 2013–2014.

Variables	Total^1^ (N = 973)	HTN prevalence		Crude PR^2^ (95.0% CI)^3^	p^4^
		%	(95.0% CI)^3^		
**Age (years)**					
12–14	472	5.9	(3.7–9.4)	1.00	
15–17	501	6.4	(3.8–10.7)	1.08 (0.48–2.42)	0.838
**Race/skin color (self-reported)**^5^					
White	348	5.1	(2.8; 9.4)	1.00	
Black	47	9.5	(3.2; 25.7)	1.85 (0.50–6.75)	0.339
Mixed	530	6.4	(4.5; 9.1)	1.24 (0.57–2.68)	0.567
Other^3^	38	7.6	(2.4; 21.7)	1.49 (0.48–4.54)	0.473
**Type of school attended**					
Public	623	6.1	(4.2; 8.7)	1.00	
Public	350	6.4	(4.0; 10.2)	1.05 (0.57–1.91)	0.873
**Term**					
Morning	783	6.4	(4.6; 8.9)	1.00	
Afternoon	190	5.2	(2.8; 9.4)	0.81 (0.40–1.63)	0.549
**Tobacco use (lifetime)**					
No	799	6.5	(4.9; 8.7)	1.00	
Yes	174	4.6	(2.0; 10.1)	0.70 (0.31–1.58)	0.386
**Tobacco use (previous 30 days)**					
No	932	6.1	(4.4; 8.4)	1.00	
Yes	33	5.1	(0.8; 27.4)	0.84 (0.11; 5.95)	0.861
**Alcohol use (lifetime)**					
No	354	5.6	(3.1; 9.7)	1.00	
Yes	574	6.3	(4.3; 9.1)	1.12 (0.53–2.38)	0.752
**Alcohol use (previous 30 days)**					
No	719	6.6	(4.8; 9.1)	1.00	
Yes	219	4.8	(2.4; 9.4)	0.72 (0.34; 1.49)	0.369
**Binge drinking(previous 30 days)**					
No	845	5.8	(4.3; 7.9)	1.00	
Yes	64	8.7	(2.5; 25.7)	1.46 (0.41–5.18)	0.545
**Physical Activity**					
Active	368	6.5	(4.2; 10.0)	1.00	
Inactive	562	6.4	(4.5; 9.2)	0.97 (0.56–1.69)	0.936
**Diabetes mellitus (self-reported)**					
No	930	6.0	(4.4; 8.2)	1.00	
Yes	40	9.9	(3.5; 25.1)	1.63 (0.54–4.94)	0.371
**Hypercholesterolemia (self-reported)**					
No	835	5.5	(4.0; 7.5)	1.00	
Yes	96	7.1	(3.2; 15.3)	1.29 (0.53–3.10)	0.558
**Nutritional status**					
Normal	747	4.6	(3.3; 6.4)	1.00	
Overweight	154	9.9	(6.1; 15.7)	2.15 (1.17–3.92)	**0.014**
Obese	54	16.4	(6.7; 34.6)	3.51 (1.52–8.12)	**0.004**

^1^Number of valid responses

^2^Prevalence Ratio

^3^95% Confidence Interval

^4^Wald’s Chi-square test

^5^Includes Native Brazilian and Asian ethnic groups.

**Table 5:** Prevalence and risk factors associated with HTN in adolescent males. ERICA Study, Goiânia, Goiás, Central-West region of Brazil, 2013–2014.

Variables	Total^1^ (N = 613)	HTN prevalence		Crude PR^2^ (95.0% CI)^3^	p^4^
		%	(95.0% CI)^3^		
**Age (years)**					
12–14	319	9.6	(5.7; 15.8)	1.00	
15–17	294	18.3	(11.8; 27.3)	1.90 (0.94–3.85)	0.071
**Race/skin color (self-reported)**					
White	249	16.9	(10.2–26.7)	1.00	
Black	42	9.2	(2.9; 25.5)	0.54 (0.14–2.03)	0.354
Mixed	287	12.1	(8.4; 17.0)	0.70 (0.38–1.30)	0.260
Other^5^	20	13.1	(3.5; 38.6)	0.77 (0.21–2.73)	0.678
**Type of school attended**					
Public	373	13.9	(9.4; 20.2)	1.00	
Public	240	14.0	(8.8; 21.7)	1.00 (0.54; 1.84)	0.982
**Term**					
Morning	514	14.8	(10.5; 20.4)	1.00	
Afternoon	99	10.0	(3.5; 25.6)	0.67 (0.22–2.03)	0.475
**Tobacco use (lifetime)**					
No	489	14.2	(10.4; 19.1)	1.00	
Yes	124	12.9	(6.5; 23.8)	0.90 (0.47; 1.70)	0.747
**Tobacco use (previous 30 days)**					
No	582	13.9	(10.1; 18.8)	1.00	
Yes	29	16.7	(6.8; 35.4)	1.18 (0.50; 2.80)	0.686
**Alcohol use (lifetime)**					
No	270	9.1	(5.3; 15.2)	1.00	
Yes	315	18.4	(12.2; 27.0)	2.01 (0.95; 4.25)	0.064
**Alcohol use (previous 30 days)**					
No	466	10.1	(7.0; 14.6)	1.00	
Yes	126	24.4	(10.6; 46.6)	2.40 (0.89; 6.49)	0.081
**Binge drinking(previous 30 days)**					
No	546	13.3	(10.0; 17.8)	1.00	
Yes	31	26.8	(10.8; 52.6)	1.98 (0.96; 4.09)	0.061
**Physical Activity**					
Active	587	14.1	(10.3; 19.0)	1.00	
Inactive	17	14.0	(4.4; 36.4)	0.97 (0.33; 2.87)	0.968
**Diabetes mellitus (self-report)**					
No	507	13.2	(9.1; 18.9)	1.00	
Yes	57	20.2	(12.1; 31.9)	1.49 (0.73; 3.02)	0.261
**Hypercholesterolemia (self-report)**					
No	341	13.3	(8.1; 21.1)	1.00	
Yes	216	16.3	(10.7; 24.1)	1.21(0.58; 2.52)	0.596
**Nutritional status**					
Normal	410	10.5	(6.0; 17.8)	1.00	
Overweight	110	21.4	(15.4; 29.1)	2.05 (1.05; 3.99)	**0.035**
Obese	73	24.9	(16.0; 36.6)	2.36 (1.16; 4.82)	**0.019**

^1^Number of valid responses

^2^Prevalence ratio

^3^95.0% Confidence Interval

^4^Wald’s Chi-square test

^5^Includes Native Brazilian and Asian ethnic groups.

**Table 6:** Analysis of the Poisson regression of factors associated with HTN, by gender. ERICA study, Goiânia, Goiás, Central-West region of Brazil, 2013–2014.

Variables	Girls			Boys		
	APR^1^	95.0% CI^2^	p^3^	APR^1^	95.0% CI^2^	p^3^
**Age (years)**						
12–14	1.00			1.00		
15–17	1.29	0.53–3.14	0.551	1.72	0.78–3.82	0.171
**Race/skin color (self-reported)**						
White	1.00			1.00		
Black	1.84	0.47–7.14	0.366	0.72	0.17–2.98	0.646
Mixed	1.36	0.57–3.25	0.476	0.80	0.44–1.45	0.461
Other^4^	1.81	0.61–5.35	0.273	0.91	0.23–3.46	0.887
**Type of school**						
Public	1.00			1.00		
Public	1.25	0.67–2.35	0.460	1.02	0.54; 1.94	0.342
**Term**						
Morning	1.00			1.00		
Afternoon	0.95	0.47–1.94	0.902	0.61	0.21–1.72	0.342
**Binge drinking (previous 30 days)**						
No				1.00		
Yes	-	-	-	1.27	0.58–2.80	0.534
**Nutritional status**						
Normal	1.00			1.00		
Overweight	2.28	1.26–4.09	**0.007**	1.85	0.76; 4.49	0.163
Obese	3.48	1.42–8.54	**0.008**	2.30	1.09; 4.84	**0.029**

^1^Adjusted prevalence ratio

^2^95.0% Confidence Interval

^3^Wald’s Chi-square test

^4^Includes Native Brazilian and Asian ethnic groups.

## Discussion

This study investigated the prevalence of and factors associated with hypertension in 1,586 adolescents from 36 schools in Goiania City, the capital of the State of Goiás, located in the Central-West region of Brazil. The results showed a 10.1% prevalence of HTN, which was statistically higher in boys than in girls. In addition, a regression analysis showed that nutritional status was the only risk factor for HTN shared by both genders. In both boys and girls, obesity was shown to be independently associated with HTN. However, being overweight was shown to be an associated factor in girls only. A high prevalence of physical inactivity was also observed in the adolescents investigated, and particularly in the girls. On the other hand, a higher prevalence of obesity was found in the boys, as compared to the girls.

The prevalence of HTN estimated in this study was similar to that found in Brazilian adolescents between 12 and 17 years old (9.6%; 95.0% CI: 9.0–10.3) [25]. It also confirmed the findings of a meta-analysis conducted among Brazilian adolescents (8.1%; 95.0% CI: 6.2–10.5%)[8]. On the other hand, a previous study of 1,375 adolescents aged 12–14 in Goiânia, the location of this study, estimated a HTN prevalence of 2.0% (95.0% CI: 1.4–2.9%) [39], a frequency lower than that found in this investigation. We believe that methodological differences and population characteristics may have contributed to these differences between the studies. The previous study included no adolescents older than 14. In addition, the prevalence of obesity was lower than that estimated in this study [39].

Other studies have shown a wide variation in estimates of theprevalence of HTN in adolescents [8]. In the Southeast Region, investigations have estimated that3.3% of adolescents have HTN (n = 487) in Ouro Preto (Minas Gerais State) [40]; 9.5% (n = 704) in Cubatão (São Paulo State) [41]; and 19% (n = 884) in Rio de Janeiro (Rio de Janeiro State) [42]. In the southern region of the country, the prevalence of HTN has been calculated at11.3% (n = 511) in Porto Alegre (Rio Grande do Sul State) [43]; 12.4% (n = 233) in Londrina (Paraná State) [44];and 12.6% (n = 497) in Curitiba (Paraná State) [45]. In the Northeast Region, the prevalence of HTN has been recorded as 7.4% (n = 674) in João Pessoa (Paraíba State) [46]; and as 17.3% (n = 1,878) in Recife (Pernambuco State) [14]. In the Midwestern Region, a prevalence of 11.7% (n = 1,716) was estimated for Cuiabá (Mato Grosso State) [47]. This great variability in the estimates of HTN prevalence may reflect significant differences in the BP measurement and classification methodologies used.

In the present study, male adolescents presented a higher prevalence of HTN than female adolescents. A previous study conducted in Goiânia found similar prevalencerates in males and females (2.8% versus 3.1%) [39]. A systematic review and meta-analysis of Brazilian studies found a statistically higher prevalence of HTN in male adolescents than in female adolescents (8.75% versus 6.31%; p < 0.001) [8]. Multiple mechanisms may contribute to this association. For example, the increase in male testosterone levels in early adolescence has a potential prohypertensive effect, increasing the prevalence of HTN [25, 48]. In addition, estrogen has the cardioprotective effect of endogenous estradiol in BP, since it is responsible for activating the vasodilator pathway mediated by nitric oxide and prostacyclin, in addition to inhibiting the sympathetic nervous system and angiotensin, thus decreasing HTN prevalence in girls[49]. In general, boys have a higher prevalence of visceral and intra-abdominal fat accumulation and obesity than girls. These factors promote increased sympathetic activity, leading to increased sodium reabsorption and peripheral vascular resistance, and contributing to HTN [25, 50]. In fact, in this study, the prevalence of HTN was statistically higher in boys than in girls.

This study observed an association between HTN and being overweight in girls and between HTN and obesity in both genders. In addition, there was a positive correlation between the SBP/SBP percentile and BMI Z-score/NS in both genders. A cross-sectional study conducted among1,716 adolescents aged 10–16 in Brazil supports our results[47]. The authors found that, after adjusting for age, gender, and race/skin color, obesity was the main factor associated with HTN[47]. In fact, it is estimated that 17.8% of the prevalence of HTN in Brazilian adolescents can be attributed to obesity [25]. Obesity is a key factor in the development of disorders such as dyslipidemia, insulin resistance, and diabetes mellitus, which in turn are associated with an increase in negative cardiovascular events such as stroke, acute myocardial infarction, and coronary heart disease [51]. The insulin resistance and inflammation caused by obesity may alter vascular function and lead to HTN[52].

This study has some limitations. First, the cross-sectional nature of the investigation does not allow the establishment of cause-and-effect relationships [53, 54]. Second, some variables (for example, medical history and tobacco and alcohol consumption) were self-reported, and therefore subject to memory and response bias[55]. Third, three blood pressure measurements were carried out by the same evaluator, which may have led to observer bias and an overestimation of HTN. In addition, no Ambulatory Blood Pressure Monitoring (ABPM) was performed to better characterize the BP profiles of the adolescent participants [56]. Fourth, it was not possible to evaluate the percentage of body fat in each participant, to better characterize his or her NS. Finally, the generalization of results has been limited [57], since only individuals enrolled in participating schools were considered, and not the population of adolescents outside this environment.

In conclusion, there was a high prevalence of HTN in the adolescents investigated, and particularly in the male participants. Obesity was identified as the main risk factor for HTN in both genders and being overweight was identified as a risk factor in girls. A high frequency of obesity was observed, especially in the male adolescents. On the other hand, high levels of physical inactivity were observed in the female adolescents. The results of this investigation reveal a need to implement strategies to prevent and control HTN and its associated risk factors in the school context. Information distributed to adolescents on beneficial health behaviors should focus on promoting good health. Thus, Institutions of Higher Education (IHEs) and Brazil’s health services should play a social role in public school spaces through effective interventions, such as encouraging healthy eating, promoting regular physical activity, and constantly observing HTN prevalence and its associated risk factors (e.g. being overweight and obesity), among other initiatives. The HEIs can collaborate in the training of education and healthcare professionals by articulating the actions of the Ministry of Health of Brazil. These actions are important, as they may lead to early detection and control of HTN, helping to prevent this condition in adult life, and decreasing the HTN load in the Brazilian population.
